# Effects of indoor biophilic environments on cognitive function in elderly patients with diabetes: study protocol for a randomized controlled trial

**DOI:** 10.3389/fpsyg.2025.1512175

**Published:** 2025-02-19

**Authors:** Jiajia Dai, Mohan Wang, Han Zhang, Zhengfang Wang, Xue Meng, Yanan Sun, Yuan Sun, Wenhui Dong, Zhiying Sun, Kuo Liu

**Affiliations:** ^1^Department of Epidemiology and Health Statistics, School of Public Health, Capital Medical University, Beijing, China; ^2^Beijing Municipal Key Laboratory of Clinical Epidemiology, Beijing, China; ^3^School of Architecture, Harbin Institute of Technology (Shenzhen), Shenzhen, China; ^4^Health Management Center, Beijing Aerospace General Hospital, Beijing, China; ^5^School of Architecture and Design, Harbin Institute of Technology, Harbin, China; ^6^Key Laboratory of Cold Region Urban and Rural Human Settlement Environment Science and Technology, Ministry of Industry and Information Technology, Harbin, China

**Keywords:** indoor biophilic elements, cognition, elderly diabetic patients, virtual reality, *APOE4*

## Abstract

**Background:**

The prevalence of cognitive impairment in elderly diabetic patients is increasing, highlighting the importance of exploring strategies to prevent and ameliorate cognitive impairment in this population. Previous studies have focused mostly on improving cognition in elderly diabetic patients through three methods: medication, cognitive training, and lifestyle intervention. However, few studies have investigated the role of indoor biophilic environments in improving cognition. Biophilic environments improve human health by integrating natural elements into indoor architectural settings and have demonstrated efficacy in reducing stress and improving cognition. Therefore, it is worth exploring the effects of indoor biophilic environments on cognition in elderly diabetic patients. This study aims to investigate the effects of indoor biophilic environments on cognition in elderly diabetic patients, and the potential mechanisms.

**Methods:**

This is a single-center, randomized controlled trial, which includes a short-term VR intervention and a long-term real environment intervention. In the short-term intervention trial, 64 diabetic patients over 60 years old are randomly assigned to 1 of 7 intervention groups or a control group. All intervention groups are constructed by single or various combinations of the 3 indoor biophilic elements: natural decorative paintings, indoor potted plants, and ornamental fish. The primary outcome of the short-term intervention trial is the cognitive scores assessed by DSST and BDS. In the long-term intervention trial, 240 diabetic patients over 60 years old will be randomly assigned to either the intervention group or the control group. The biophilic elements in long-term intervention will be determined based on the results of the short-term VR intervention trial. The primary outcomes of the long-term intervention trial are cognitive scores measured by DSST and MoCA, as well as concentrations of plasma p-tau181, esRAGE, and IL-6.

**Conclusion:**

The findings will be utilized to develop a restorative living environment for elderly patients with diabetes to improve cognition.

**Clinical trial registration:**

https://www.chictr.org.cn, identifier [ChiCTR2300072329].

## 1 Introduction

Diabetes represents a global public health concern, posing an increasingly formidable challenge to healthcare systems worldwide. In 2021, the global age-standardized prevalence of diabetes reached 6.1%. Within the age range of 65–95 years, the prevalence of diabetes exceeded 20% in every five-year age interval (Ong et al., 2023). Cognitive impairment is a significant complication of diabetes and manifests as a decline in cognitive abilities related to diabetes, as well as mild cognitive impairment (MCI) and dementia (Biessels and Despa, 2018). Diabetes, cognitive impairment, and dementia often coexist within people older than 65 years (Srikanth et al., 2020). Approximately 26% of people over 60 with diabetes have MCI and 36.9% have dementia (Jia et al., 2020). Cognitive impairment weakens the ability of self-management in diabetes, while inadequate diabetes management increases the risk of cognitive dysfunction (Ojo and Brooke, 2015). Previous research also indicated that more severe cognitive impairment related to diabetes predominantly occurs in older people (Biessels and Despa, 2018). Elderly diabetes patients with coexisting cognitive impairment experience a significant reduction in quality of life because they face difficulties in performing diabetes self-care (American Diabetes Association Professional Practice Committee, 2024; Munshi, 2017). Since 2017, the American Diabetes Association has recommended screening for cognitive impairment in elderly diabetes to enhance diabetes self-management and quality of life (Sonne and Hemmingsen, 2017).

Previous studies have explored various approaches to improve cognitive function in diabetic patients: pharmacological interventions (Cukierman-Yaffe et al., 2020; Li et al., 2021; Parish et al., 2022; Ryan et al., 2006), cognitive training (Silverman et al., 2023; Wong et al., 2020), and lifestyle interventions (Chen Y. N. et al., 2023; Espeland et al., 2018; Kwok et al., 2017; Lotan et al., 2021). Some of the antidiabetic drugs had positive effects on cognitive function (Cukierman-Yaffe et al., 2020; Li et al., 2021; Ryan et al., 2006), however, the effects of pharmacological interventions are largely limited by the patients’ compliance (Chen P. C. et al., 2023; Zullig et al., 2019). Moreover, lifestyle interventions, such as increased physical activity can also improve cognitive performance in diabetic patients (Chen Y. N. et al., 2023). However, it is difficult for the patients to maintain healthy behaviors, because they often passively alter their habits through health behavior interventions (Deslippe et al., 2023).

Though physical activities (Rivera Miranda et al., 2024) and social engagement (Liebzeit et al., 2022; Parra Rizo and López Marin, 2020) have been found to contribute to cognitive health in older adults. However, maintaining long-term physical activities and engagement with social activities is a challenge for older adults due to physical limitations or lack of motivation (De Coninck et al., 2021; Franco et al., 2015). Similarly, although many studies have confirmed the positive effects of outdoor natural environments on cognition (Besser, 2021; Bratman et al., 2012; Chen et al., 2022; De Keijzer et al., 2018), the adaptive capacity and physical function of elderly individuals gradually decline with age. Consequently, they spend more time indoors, with limited exposure to natural environments (Lee and Park, 2021). It has been demonstrated that patients after cholecystectomy whose hospital room windows faced natural landscapes had shorter postoperative hospital stays, received fewer negative evaluations during their stay, and required less potent analgesics than patients with windows facing brick walls of the building (Ulrich, 1984). The study showed that even without direct contact with the outdoor natural environments, the indoor environments still have healing effects. Biophilic design aims at incorporating natural elements into interior design, thereby enhancing human connection with nature (Barbiero and Berto, 2021). Compared to pharmacological and lifestyle interventions, biophilic design improves cognitive function by creating accessible and sustainable environments that improve psychological and physiological responses (Felly and Susanto, 2020), with lower patient compliance requirements. Recent studies have demonstrated that biophilic design has potential benefits on cognition (Mostajeran et al., 2023; Wallmann-Sperlich et al., 2019; Yin et al., 2019), but few have shown which specific biophilic elements are most beneficial to cognition.

Constructing a therapeutic environment that incorporates various biophilic design elements in a real indoor environment is a challenging task, as the optimal biophilic elements remain ambiguous. Virtual reality (VR) technology can simulate the real environment and integrate various sensory stimuli (Riva et al., 2020), rendering the exploration of optimal biophilic elements or their combinations more cost-effective. In recent years, an increasing number of studies have utilized VR technology to construct biophilic environments and demonstrated its beneficial effects on cognitive performance (Latini et al., 2024; Yin et al., 2019). Thus, we first use VR technology to create biophilic environments with various biophilic elements and conduct a short-term trial to screen for the most effective biophilic elements or combinations. However, VR environments also have some limitations. Virtual environments have a side effect of motion sickness which will cause a biased evaluation of healing effects (Kiryu and So, 2007) and VR cannot replicate tactile sensations (Yu et al., 2019), which may miss the healing effects of touching biophilic elements (Hassan and Zhang, 2024; Jenkins, 1986). Additionally, conventional VR technology cannot mimic the comprehensive healing effect of biophilic elements. For example, it cannot simulate the air purifying effect of potted plants (Pettit et al., 2018) and the emotional support of companion animals (Amiot and Bastian, 2015; Liu et al., 2024; Acquadro Maran et al., 2022). In addition, VR also cannot simulate the effect of reducing sedentary time and increasing physical activity caused by watering plants (Dogra et al., 2017). Therefore, referring to the healing effect of each biophilic element in the short-term trial, appropriate biophilic elements will be selected for long-term indoor environment intervention. The specific arrangement of biophilic elements in the real environment will be designed to closely replicate the layout in the VR environment, ensuring the effects of the biophilic elements.

The mechanisms underlying the impact of biophilic environments on cognition are not yet clear. A study suggests that biophilic environments may reduce the occurrence of neuroinflammation by alleviating stress, thereby improving cognition (Valentine et al., 2024), which is crucial in mitigating cognitive impairment associated with the E4 variant of apolipoprotein E (*APOE4*). *APOE4* is a strong genetic risk factor for cognitive impairment (Montagne et al., 2020). Previous studies have examined gene–environment interactions between *APOE4* and other environmental risk factors on cognitive functions. These studies mainly explored the interactions between diet (van de Rest et al., 2016), residential greenness (Zhu et al., 2020), physical activity (Pa et al., 2022; Stringa et al., 2020), and socioeconomic position (Frank et al., 2021) with *APOE4*. Investigating the interaction between *APOE4* and indoor biophilic elements on cognitive function helps us determine if biophilic interventions have different effects on populations with diverse genetic backgrounds.

Through the integrated approach of short-term VR and long-term real-environment interventions, we aim to explore the effects of indoor biophilic environments on cognitive function in elderly patients with diabetes. We use the results from the short-term VR trial as a reference for the long-term real environment intervention trial. The biophilic elements or combinations with the largest effect size and *p* < 0.1 in the short-term VR trial will be used in real indoor environments to validate their long-term effects on cognition in elderly diabetes. The findings of this research will not only contribute to supporting the design of VR scenarios for assessing the restorative impact of biophilic residential environments but also help develop cognitively improving living environments for elderly diabetic patients.

## 2 Methods

### 2.1 Design and ethics

**Figure 1:**
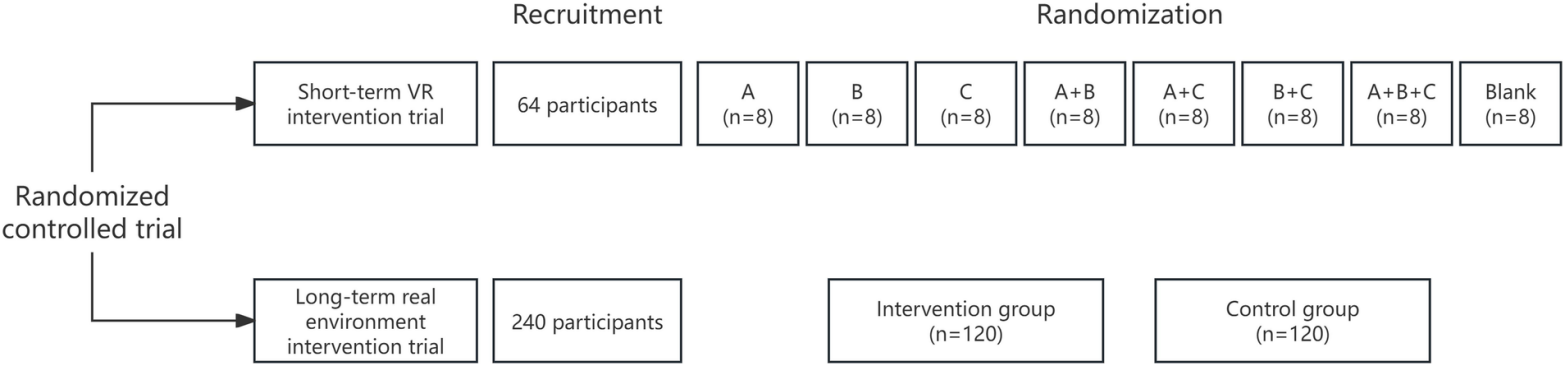
Study flow chart. VR, virtual reality; A, natural decorative paintings; B, indoor potted plants; C, ornamental fish.

This study is a single-center, randomized controlled trial. It includes both a short-term VR intervention and a long-term real environment intervention. The study flowchart is depicted in Figure 1.

As for the short-term VR intervention trial, a total of 64 participants over 60 years old with diabetes are recruited. The participants are randomly assigned to seven intervention groups or a control group using block randomization with randomly selected block sizes. The main objective of the short-term trial is to determine which biophilic elements or their combinations show the most significant improvement in cognitive function.

As for the long-term real environment intervention trial, a total of 240 participants over 60 years old with diabetes will be recruited. The participants will be randomly assigned in a 1:1 ratio to either the intervention or control group. The main objectives of this long-term trial are to provide evidence for the healing effects of biophilic environments on cognition, and whether the *APOE4* genotype will modify the healing effects of biophilic environments on cognition in elderly diabetes.

Enrollment completion and final study visit are projected for December 2024 and December 2026, respectively.

This study was approved by the Ethics Committee of Capital Medical University (Approval No: Z2023SY019) and is conducted according to the ethical standards of the Helsinki Declaration. The study protocol was developed according to the Standard Protocol Items: Recommendations for Interventional Trials (SPIRIT) checklist.

### 2.2 Study setting

This study is conducted in a subdistrict in Fengtai District, Beijing.

### 2.3 Participants recruitment

#### 2.3.1 Short-term VR intervention trial

Participants are recruited at a subdistrict in Fengtai District, Beijing. The short-term trial project is promoted to the community population in two ways: (1) to conduct a seminar on the prevention and treatment of complications in diabetic patients in the community and introduce the project; (2) to introduce the project to diabetic patients who have their regular physical examinations or visit endocrine clinics in the subdistrict.

Patients who are interested and meet the criteria are asked to sign their contact information and initially agree on a date for the VR intervention. The participants are called 2 days before the date they have agreed on and asked to provide medical records when they come to participate in the trial.

#### 2.3.2 Long-term real environment intervention trial

Participants will also be recruited at the subdistrict in Fengtai District, Beijing. Researchers will introduce the program to diabetic patients who have regular physical examinations or visit endocrine clinics in the subdistrict. Eligible patients who agree to participate in the trial will be asked to sign an informed consent form and witnessed by a member of our research team.

### 2.4 Inclusion criteria and exclusion criteria

#### 2.4.1 Short-term VR intervention trial

Participants are eligible if they (1) are aged 60 years or older, (2) have been diagnosed with diabetes by a secondary or tertiary hospital or regularly take antidiabetic drugs, and (3) signed the informed consent form.

Participants are excluded if they (1) have been diagnosed with severe mental disorders, such as schizophrenia or hysterical psychosis; (2) have been diagnosed with dementia, cognitive impairment, severe physical disabilities such as paralysis, severe mobility impairments, or conditions that require full assistance for daily living, or language barriers that hinder their ability to successfully complete the questionnaire and physical examination; (3) have cognitive impairment resulting from various neurological disorders, including Parkinson’s disease, multiple sclerosis, traumatic brain injury, stroke, epilepsy, and intracranial space-occupying lesions.

#### 2.4.2 Long-term real environment intervention trial

In addition to the inclusion and exclusion criteria described for the short-term VR intervention trial, the long-term real environment intervention trial also has some additional exclusion criteria. Participants will be excluded from the long-term trial if they (1) have large size biophilic decorative paintings (larger than 40 cm × 60 cm) in their living room; (2) have more than 2 pots of plants in their homes, and care for these plants themselves; (3) have animals such as ornamental fish in their homes, and care for these animals themselves.

### 2.5 Intervention

#### 2.5.1 Short-term VR intervention trial

**Figure 2:**
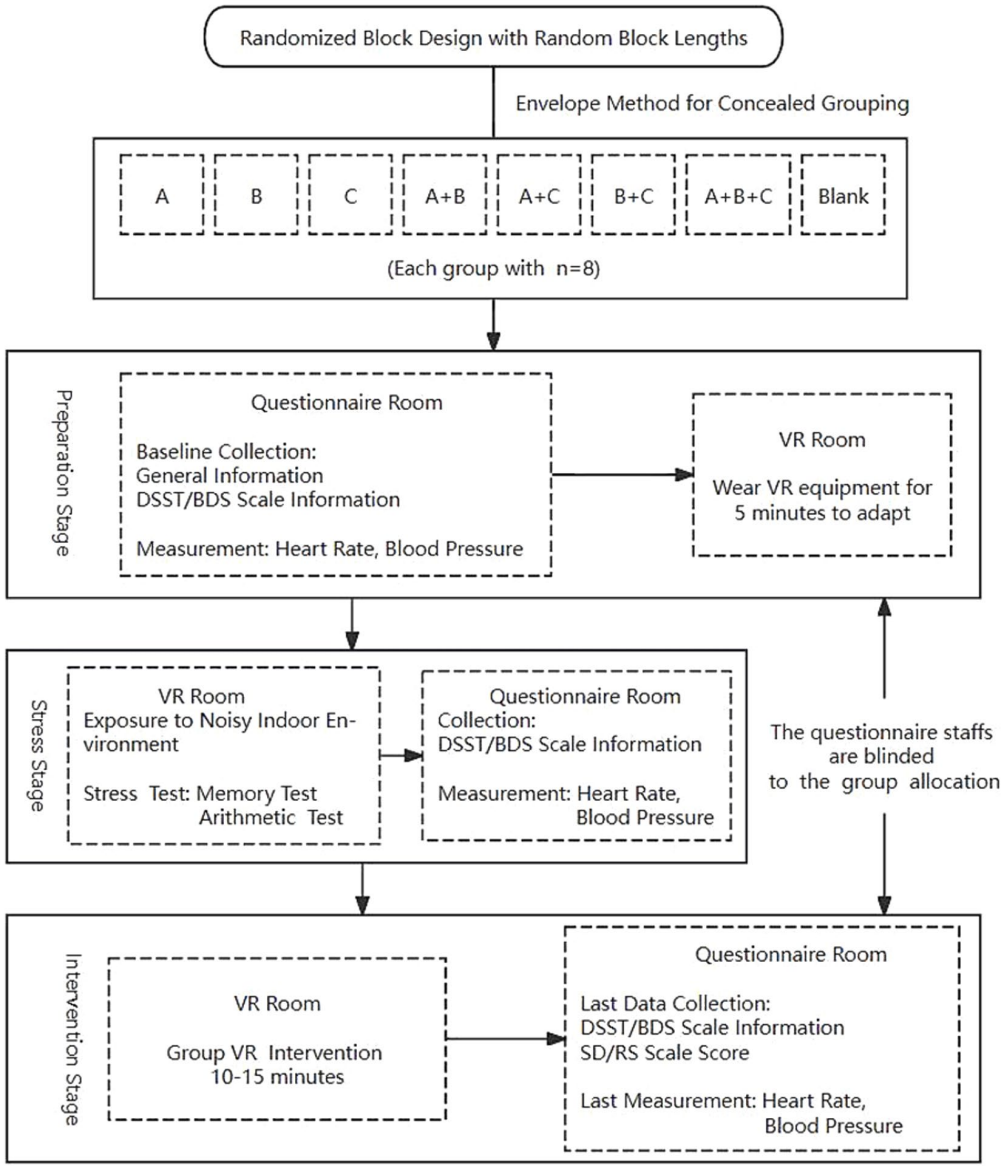
Short-term trial flow chart. A, natural decorative paintings; B, indoor potted plants; C, ornamental fish; VR, virtual reality; DSST, the Digit Symbol Substitution Test; BDS, the Backward Digit Span; SD, the Environmental Semantics Differential Scale; RS, the Restoration Scale.

The VR residential environment used in this study includes three elements: natural decorative paintings (A), indoor potted plants (B), and ornamental fish (C). Biophilic design encompasses three patterns: nature in the space, natural analogues, and nature of the space (Hung and Chun, 2021). Current research primarily focuses on the first two patterns and has found that biophilic elements such as plants (Verzwyvelt et al., 2021; Yin et al., 2020), animals (Motooka et al., 2006), and natural decorations (Shen et al., 2020) help alleviate stress and improve cognitive function. Considering the lifestyle habits of local older adults and the feasibility of practical implementation, our study employs the three biophilic elements. A blank control VR scene without biophilic design elements and seven biophilic design VR scenes (A, B, C, A + B, A + C, B + C, A + B + C) are employed. The experiment follows the subsequent steps. The trial flowchart is illustrated in Figure 2.

- Preparation Phase: first, researchers provide a concise introduction to the study procedures. Participants rest for 5 min, and heart rate and blood pressure are measured using an Omron medical electronic blood pressure monitor (HBP-1300). Then, baseline data are collected through a questionnaire survey. The structured questionnaire includes general demographic characteristics such as age, gender, height, weight, ethnicity, education level, marital status, and income. It also includes lifestyle behaviors such as smoking, alcohol consumption, and physical activity, medical histories such as hypertension, diabetes, and cardiovascular disease, and medication history. An assessment of mental health status is conducted using the Center for Epidemiologic Studies Depression Scale (10-item, CESD-10). Finally, cognitive scores are assessed using the Digit Symbol Substitution Test (DSST) and the Backward Digit Span (BDS) scale. After the measurements, researchers provide the VR headset to participants and allow them to adapt and learn basic operations for up to 5 min. All VR devices look the same shape and color.
- Stress Phase: participants are exposed to a cluttered living environment through VR, with background noise from bustling traffic and mechanical devices. Then they undergo stress-inducing tests, including a memory test and an arithmetic test. The stress phase lasts for 5 min. After this phase, cognitive scores (DSST/BDS), heart rate, and blood pressure are re-measured.
- VR Intervention Phase: participants will engage in a 10–15 min of VR scenario intervention according to their group allocation. Depending on their groups, some participants may simulate activities such as walking indoors, opening doors, pulling curtains, watering plants, or feeding fish. After completing the VR intervention, cognitive scores (DSST and BDS), heart rate, and blood pressure are measured again. Additionally, the Environmental Semantics Differential Scale (SD) and the Restoration Scale (RS) are also evaluated.

During the VR experience, researchers record participants’ motion sickness. If participants feel nauseous or dizzy, they are instructed to remove the VR headset and take a two-minute break. Those who recover resume the trial, whereas those who remain symptomatic are withdrawn from the study.

##### 2.5.1.1 Virtual environments

VR interventions are deployed on the HTC Cosmos head-mounted display. The HTC Cosmos is a standalone device equipped with a high-resolution display that provides clear and realistic images. It also features built-in speakers and a microphone. The HTC Cosmos is equipped with a head tracking system and independent hand controllers, which enables users to perform various actions in VR, such as grabbing, moving, and rotating objects, enhancing immersion and interactivity.

We created nine distinct virtual residential environments for participants to explore and interact with, utilizing various 3D models and materials within the Unity game engine (version 2022.3.2f1c1). These VR scenes are arranged in the indoor living room and bedroom. The indoor residential environments serve as experimental scenarios, including a cluttered group (a cluttered house with traffic noise and no biophilic elements), a blank control group (a tidy house without any biophilic elements designed, blank), and seven intervention groups with a biophilic environment consisting of natural decorative paintings (A), indoor potted plants (B), ornamental fish (C) and their combinations (A + B, A + C, B + C, A + B + C).

The virtual environments are displayed on the HTC Cosmos head-mounted display (with a single-eye resolution of 1,440 × 1,700 pixels and a total resolution of 2,880 × 1,700 pixels; refresh rate of 90 Hz), and integrated headphones and controllers are used. To render the virtual environments, we utilize computers with a Windows 10 desktop system (NVIDIA GeForce RTX 2080 SUPER, Intel Core i7-8700K, 3.70 GHz).

#### 2.5.2 Long-term real environment intervention trial

**Figure 3:**
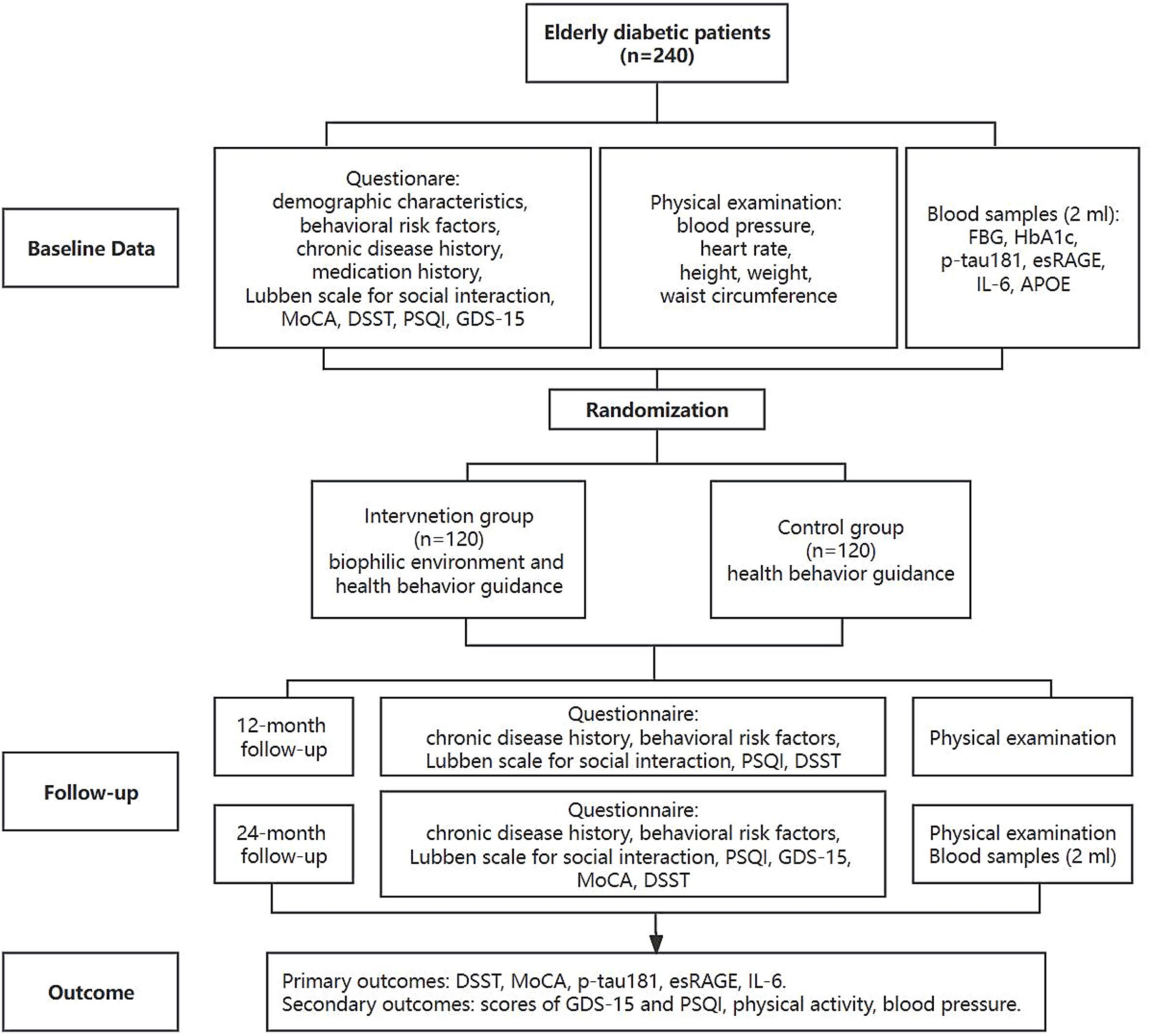
Long-term trial flow chart. MoCA, the Montreal Cognitive Assessment scale; DSST, the Digit Symbol Substitution Test; PSQI, the Pittsburgh Sleep Quality Index; GDS-15, 15-item Geriatric Depression Scale; FBG, fasting blood glucose; HbA1c, glycated hemoglobin; p-tau181, tau phosphorylated at threonine 181; esRAGE, endogenous secretory receptor for advanced glycation end-products; IL-6, interleukin-6; *APOE*, E4 variant of apolipoprotein E.

The trial flowchart is depicted in Figure 3.

At baseline, after the participants sign the informed consent form, they will be administered a questionnaire to collect the following information: demographic characteristics (sex, date of birth, education), behavioral risk factors (dietary habits, smoking, alcohol consumption, physical activity, etc.), chronic disease history, medication history, the Lubben scale for social interaction, the Montreal Cognitive Assessment (MoCA) scale, DSST, the Pittsburgh Sleep Quality Index (PSQI), and the 15-item Geriatric Depression Scale (GDS-15). After completing the questionnaire, participants will undergo a physical examination (including blood pressure, heart rate, height, weight, and waist circumference), and then 2 mL blood samples will be collected by venipuncture. Then, researchers will distribute the wGT3x-BT accelerometer (Actigraph) to part of the participants. The wGT3x-BT accelerometer will objectively collect data regarding physical activity and sleep patterns, including time spent in light, moderate, and vigorous physical activity, sedentary time, sleep duration, and sleep efficiency. After completing the aforementioned steps, envelopes will be opened to decide which group the subjects would be enrolled in.

Both the intervention and control groups will receive health behavior guidance, while the intervention group will additionally receive an indoor biophilic environment intervention. Health behavior guidance includes personalized health behavior and dietary guidance based on the Guideline for the prevention and treatment of type 2 diabetes mellitus in China (2020 edition) (Zhu and Society, 2021). Health promotion and personalized guidance will be conducted at each follow-up visit by medical doctors or professionals. The biophilic elements that are most effective in enhancing cognition during the short-term VR intervention trial will be used as the biophilic interventions in the long-term real environment intervention trial. Participants in the intervention group will be told to take the biophilic elements (such as indoor plants) home and place them in a visible place.

Long-term interventions will be followed up at 12 and 24 months. At the 12-month follow-up, behavioral risk factors, chronic disease history, Lubben scale for social interaction, PSQI, and DSST will be collected and a general physical examination will be performed. At the 24-month follow-up, behavioral risk factors, chronic disease history, Lubben scale for social interaction, PSQI, GDS-15, cognitive function (DSST, MoCA) will be assessed, a general physical examination will be performed, and blood samples will be collected again.

Blood samples will be collected in ethylenediaminetetraacetic acid (EDTA) anticoagulated vacutainer tubes. After centrifugation, blood cells and plasma will be stored separately. Plasma samples will be used for biomarker detection at both baseline and the 24-month follow-up, including glycated hemoglobin (HbA1c) levels, fasting blood glucose (FBG), tau phosphorylated at threonine 181 (p-tau181), endogenous secretory receptor for advanced glycation end-products (esRAGE), and interleukin-6 (IL-6). Blood cells will be used for DNA extraction and *APOE* genotyping. Blank control and duplicate samples will be used for quality control during the experiment.

At baseline and follow-up visits, we will fill out registration forms to record the use of the biophilic intervention facilities in the living room, in order to replace damaged biophilic elements in time and to provide persistent biophilic indoor environment intervention.

### 2.6 Withdrawal

Participation is voluntary, and participants can withdraw consent at any time. Reasons for withdrawal will be documented.

#### 2.6.1 Short-term VR intervention trial

In the event of any discomfort, including dizziness or nausea, experienced by a participant during the VR intervention, the intervention is promptly terminated. The participant is then given a two-minute break for recovery. If the discomfort continues post-rest, the participant is advised to withdraw from the study after a consultation.

#### 2.6.2 Long-term real environment intervention trial

Being lost to follow-up does not constitute withdrawal from the study. Research staffs will make efforts to re-engage participants for future assessments. For instance, if a participant is unreachable at the 12-month check-in, this is noted as a missed appointment, and attempts will be made to reach them for the 24-month follow-up.

### 2.7 Blinding

#### 2.7.1 Short-term VR intervention trial

Researchers from the survey group collect epidemiological data and assess participants’ cognitive function through questionnaires. Survey group researchers are blinded to the group allocation. The VR intervention process cannot be completed independently by participants. VR operators need to assist in the procedure and guide participants to fully engage with the VR scene. Therefore, it is not feasible to blind either the participants or the VR operators. To minimize bias, cognitive outcome assessments will be conducted by survey group researchers who are not involved in the VR intervention.

#### 2.7.2 Long-term real environment intervention trial

Since biophilic elements are easily identifiable, only questionnaire administrators, outcome assessors, and data analysts will be blinded to the group allocation.

### 2.8 Outcomes

#### 2.8.1 Short-term VR intervention trial

1. Primary outcome: cognitive function, evaluated using the DSST and BDS scales.
2. Secondary outcome: scores of the SD and the RS scales, as well as blood pressure and heart rate.

#### 2.8.2 Long-term real environment intervention trial

1. Primary outcome: cognitive function assessed by the DSST and MoCA scales, as well as concentrations plasma of p-tau181, esRAGE and IL-6.
2. Secondary outcome: the amount of physical activity, blood pressure, scores of GDS-15 and PSQI. Participants will be asked to report the number of days per week they engage in vigorous physical activity (VPA), moderate physical activity (MPA), and walking, along with the daily duration of these activities. The metabolic equivalents (METs) of each study subject will be assigned and calculated based on the above information (Deng et al., 2008), and the amount of physical activity is the sum of METs.

### 2.9 Measurements of cognitive function

#### 2.9.1 Short-term VR intervention trial

The DSST and BDS tests have been widely used in the field of VR environments for cognitive function, and they are highly responsive to the immediate effects of short-term interventions (Liu et al., 2022; Yin et al., 2018). Therefore, cognitive function is assessed by DSST (score range: 0–90) and BDS (score range: 0–9) scales. The DSST test is a simple and fast assessment tool that measures the ability to link symbols with numbers in 90 s (Beres and Baron, 1981). The DSST score is the number of symbols correctly filled in consecutively within 90 s. In the BDS scale, testing begins with three-digit numbers, which are read aloud to the participant, who is then required to repeat them in reverse. The test progresses sequentially to nine-digit numbers, with each sequence allowing up to two attempts. If a participant fails to correctly repeat a sequence of a specified length twice consecutively, the test is stopped. The number of digits in the longest correctly repeated sequence serves as the measure of cognition.

#### 2.9.2 Long-term real environment intervention trial

Cognitive scores will be derived from the DSST and MoCA scales (score range: 0–30). The MoCA can track gradual, multidimensional cognitive decline over extended periods, complementing DSST’s focus on processing speed—a domain particularly impaired in diabetes-related cognitive dysfunction (Li et al., 2023; Rawlings et al., 2015). The DSST score measurement is the same as in the short-term trial. The MoCA is a brief cognitive screening tool with high sensitivity in detecting MCI (Nasreddine et al., 2005). It measures several cognitive domains, including attention, memory, delayed recall, language, executive function, abstraction, and orientation to time and place (Bischoff-Ferrari et al., 2020). The MoCA consists of 30 questions, and the score is equal to the number of correct answers out of the 30 items. If the individual reports 12 years of education or less, an additional point is added.

### 2.10 Randomization

Randomization is conducted by a computer-generated list of random numbers made by independent statisticians without any clinical involvement in the study. The block randomization sequence is generated using SAS 9.4 software (PROC PLAN). The randomized block sizes are set to 8 and 16 for the short-term VR intervention trial and 2 and 4 for the long-term real environment intervention trial. After obtaining eligible patient consent, allocations are determined using sequentially numbered sealed envelopes containing information disclosing the type of intervention to be applied. Additionally, group assignments will be concealed until the intervention begins.

### 2.11 Sample size

#### 2.11.1 Short-term VR intervention trial

The randomized block analysis of the variance module of PASS 11.0 was used to calculate the sample size. According to previous literature (Liao et al., 2020), the MoCA scores would increase by 0.5 points, and the Chinese version of the Verbal Learning Test (CVVLT) immediate recall scores would increase by 1.0 point after VR intervention. The DSST has been found to be more sensitive than the MoCA and CVVLT. Thus, we conservatively assumed that the mean difference in DSST scores between the intervention and control groups would be 1.0, with a standard deviation of 1.2. The number of blocks was set at 8, the significance level was set at *p* < 0.05 and the statistical power was set at 90%. Based on the above parameters, the minimum sample size per block is six individuals. Considering the possibility of noncompliance or refusal to participate, the sample size was increased by 20%, resulting in eight individuals per block. Thus, there are eight blocks in the short-term trial, with each block containing eight individuals.

#### 2.11.2 Long-term real environment intervention trial

The randomized block analysis of the variance module of PASS 11.0 was used to calculate the sample size. Due to the lack of long-term longitudinal studies on the impact of biophilic living environments on the MoCA scores in elderly diabetic patients, we referred to studies on the impact of natural environments on cognitive scores. Changes in cognitive scores ranged from 0.020 to 0.029 for each interquartile range shift in the Normalized Difference Vegetation Index (NDVI) (De Keijzer et al., 2018). Based on these findings, we set the between-group difference in cognitive scores at 0.02, with a standard deviation of 0.017, an intraclass correlation coefficient of 0.02, and the number of blocks was set at 2. With these parameters, achieving a statistical power of 90% at the 0.05 level of two-sided significant difference requires 99 participants per group. Accounting for about 20% loss to follow-up, each group will initially need to enroll at least 120 participants, totaling 240 participants across both intervention and control groups. Considering that our participants are older adults, the likelihood of noncompliance is higher than others. Therefore, we chose a 20% non-compliance rate to ensure the study has sufficient statistical power.

Furthermore, the logistic regression module of PASS11.0 was used to calculate the sample size required for analyzing the association between the *APOE4* genotype and cognitive function. According to previous research, the prevalence of MCI in Chinese people aged 60 years and older is about 15% (Jia et al., 2020), the odds ratio of the association between *APOE4* and cognitive impairment in the Chinese population was about 2.5 (Jiang et al., 2017), the *R*^2^ of *APOE4* with other covariates, such as age and gender, is about 0.05, with the significance level was set at 0.05. Based on these parameters, achieving a power of 90% in a two-sided test requires 103 participants. In summary, the long-term trial will include at least 120 subjects in each group.

### 2.12 Statistical analysis

#### 2.12.1 Short-term VR intervention trial

The analysis will be carried out according to the intention-to-treat principle. Given the short duration of this trial, missing data is expected to be minimal. Therefore, a single imputation will be used to process the incomplete data. Outliers will be identified using the interquartile range (IQR) method, defined as values below Q1 − (1.5 × IQR) or above Q3 + (1.5 × IQR), and will be replaced with Q1 − (1.5 × IQR) and Q3 + (1.5 × IQR), respectively. One-way analysis of variance (ANOVA) (for continuous and normally distributed variables) and chi-square test (for categorical variables) will be conducted to assess the baseline equivalence between the eight groups. If there are differences in characteristics between groups at baseline, the differences will be adjusted by performing multiple linear regression. ANOVA will be used to compare whether there are statistically significant differences in cognitive scores, heart rate and blood pressure among the eight groups. For each group, paired *t*-tests will be conducted to examine whether changes in cognitive scores, heart rate, and blood pressure are statistically significant between baseline, pre-intervention, and post-intervention. The identification of optimal biophilic elements will follow a two-stage analytic protocol. First, multiple linear regression models adjusted for covariates will be fitted to compare post-VR intervention cognitive scores between each intervention group and the control. Groups achieving a covariate-adjusted *p* < 0.1 in these pairwise comparisons will be considered statistically promising. Subsequently, among these candidate groups, the intervention demonstrating the largest adjusted mean difference in cognitive scores relative to the control group will be selected for long-term real environment intervention. Multiple linear regression will be used to evaluate the cognitive improvement in each group after adjusting for covariates. Continuous variables will be assessed for normality prior to analysis. If the data are not normally distributed, the Kruskal-Wallis H test will be used to compare the differences between the eight groups.

#### 2.12.2 Long-term real environment intervention trial

The modified intention-to-treat population (mITT) will be used for statistical analysis. Missing values will be imputed using multiple imputation. Firstly, two sample t-tests (for continuous and normally distributed variables) and chi-square test (for categorical variables) will be conducted to assess the baseline equivalence between the intervention and control groups. Two sample t-tests will be used to compare whether cognitive scores are statistically different between two groups. Multiple linear regression will be used to demonstrate whether the intervention is statistically associated with cognitive scores after adjusting for potential confounders. Repeated measures ANOVA will be used to compare cognitive scores across groups. The potential confounders will be corrected in the mixed-effect model to determine the association between intervention and cognitive scores. If the intervention has a direct effect on cognitive function, longitudinal causal mediation analysis will be further used to clarify the mediating factors through which the intervention may affect cognition.

To explore the interaction of *APOE4* and the biophilic environment on cognition, we will perform stratified analysis according to groups to clarify the main effects of *APOE4* on cognitive function. Then, mixed models will be used to adjust for potential covariates and to elucidate the main effect of *APOE4* on cognitive function in each group. Finally, the interaction term between the *APOE4* and the intervention group will be included in the mixed-effects model to identify the effect of the genetic-environmental interaction on cognitive function.

In our study, all statistical tests are two-tailed, with a significance level of *p* < 0.05.

### 2.13 Data management

Data collected from participants will remain confidential. To ensure participant confidentiality, all personal identifying information will be securely stored separately from the study data, and access will be restricted to authorized personnel only. All data will be scrutinized and double-entered by the researchers using EpiData 3.1 to ensure accuracy. After verification, the data will be imported into SAS 9.4 for cleaning and storage.

### 2.14 Quality control

We developed investigation manuals and provided standardized training for all personnel involved in the project, including VR operators, data collectors, clinicians, and laboratory staffs. Sequentially numbered opaque sealed envelopes are used for allocation concealment, and epidemiological data collectors will be blinded to mitigate information bias. After the face-to-face questionnaire survey, quality control procedures, including preliminary variable range and logical error checks, are conducted at the investigation site. Make sure the age of the subjects is accurate by checking the date of birth on their ID cards. In order to ensure the accuracy of diabetes diagnosis, we will further check the medical records or medication records of the self-report diabetes.

#### 2.14.1 Short-term VR intervention trial

To ensure that participants’ attention is maximally directed towards the biophilic elements during the VR experience, VR operators explain the VR environment to participants and guide them in observing the biophilic elements during the experience.

#### 2.14.2 Long-term real environment intervention trial

Biophilic intervention elements will be mailed to subjects, and project staffs will call them to confirm that they have received the intervention items. Usage instructions for the biophilic elements will be dispatched via cell phone message. Monthly follow-up will be conducted to ensure the maintenance of biophilic elements. Dead plants, animals, or damaged indoor decorations will be replaced to sustain the biophilic intervention.

A total of 2 mL whole blood sample will be collected by nurses with EDTA anticoagulant tubes. The blood samples will be centrifuged at room temperature at 1,500 rpm for 10 min. The separated plasma and blood cells will be stored in clearly labeled cryovials, respectively. Plasma and blood cells samples will be preserved in an ultra-low temperature freezer at −80°C. Plasma samples will be used for the quantitative detection of serum biomarkers (p-tau181, esRAGE, IL-6). Blood cells will be used for DNA extraction. Blank and positive controls will be incorporated into the experimental design to monitor non-specific reactions and reagent contamination.

To ensure follow-up rates, the following measures will be implemented: first, fully explain the purpose and procedures of the study to subjects during the recruitment phase. Second, participants will be asked to provide at least two types of contact information, including a mobile phone number, a fixed-line phone number, or a WeChat account. If participants do not reply during weekdays, attempts will be made on weekends. Third, appropriate incentives will be provided to the participants, including subject compensation and free health consultations offered by neurologists from tertiary hospitals.

### 2.15 Safety

The anticipated risk to participants in this study is lower than that of routine physical examinations. Participants will be instructed to remove the VR headset if they have any adverse effects or discomfort.

## 3 Discussion

Diabetes has become an increasingly critical public health problem, with a rising prevalence and high rates of disability and mortality. Moreover, diabetes is a major risk factor for cognitive impairment (Cukierman-Yaffe et al., 2020). Approximately 26% of people over 60 with diabetes suffer from MCI, and 36.9% have dementia (Jia et al., 2020). Cognitive impairment not only diminishes quality of life but also hinders diabetes management (Ojo and Brooke, 2015). Consequently, it is necessary to explore effective interventions to curb the progression of cognitive impairment in the elderly diabetic population.

Current researches primarily focus on conventional glucose-lowering treatments (Cukierman-Yaffe et al., 2020; Li et al., 2021; Ryan et al., 2006), cognitive training (Silverman et al., 2023; Wong et al., 2020), dietary nutrition (Lotan et al., 2021), and physical exercise (Chen Y. N. et al., 2023). Each of the four methods mentioned above has positive effects on cognitive function in diabetic patients. A common problem with these approaches is that their effectiveness depends on patient adherence. Biophilic design, a method of natural intervention, solves the problem of patient compliance and compensates for the shortcomings of the above methods. An increasing number of studies investigate the effects of biophilic design in indoor environments on health conditions (Lei et al., 2021; Li et al., 2022; Yin et al., 2019). However, most of these studies have focused on incorporating green vegetation, with most biophilic interventions involving short-term exposure through VR environments. Few studies have explored long-term interventions in real environments. In our study, we will evaluate diverse indoor biophilic elements, including both biophilic decorations and ornamental fish. Our study aims to identify the most effective biophilic elements or combinations for improving cognitive function by VR technology in elderly diabetic patients, and then use optimal biophilic elements for long-term real indoor environmental interventions. The findings from this study will be utilized to design a healing living environment in this population to effectively protect cognitive function. Furthermore, these findings could be applied to the design of biophilic environments in hospitals, nursing homes, and even private homes, providing therapeutic spaces that promote cognitive health. This practical application of biophilic design could help guide clinicians, architects, and healthcare providers in creating spaces that optimize cognitive health outcomes.

This study has the following strengths. First, the VR scene is designed based on a standard one-bedroom house displayed on a real estate platform in the project’s implementation area. Experiencing scenes close to real life in VR environments can weaken the bias of sensory experience caused by the strangeness of the virtual environments, and make the effect estimation of biophilic intervention more realistic and reliable. Second, the VR scene, including diverse biophilic design elements and interactive operations such as watering plants, feeding fish, opening doors, and pulling curtains, which provide a fully immersive VR experience, increases the authenticity of the VR environments and can maximize the simulation of the effect of the real environment intervention. Third, VR devices may impact cognitive function regardless of the specific VR intervention scene. To address this problem, cognitive tests will be conducted after participants use VR devices to experience both cluttered and clean environments. This approach may enable us to effectively control for potential confounding factors caused by the VR devices. Fourth, our study has both a short-term VR intervention and a long-term real environment intervention, which perfectly addresses the limitations of VR compared to real environments. Finally, since *APOE4* is the strongest genetic predisposition factor for cognitive impairment, exploring its interactions with the biophilic environment could provide evidence for formulating personalized interventions.

This study also has several limitations. First, the study is conducted in a single center and focuses only on elderly diabetics, which limits the generalizability of the findings. Second, the VR scene contains many furnishings, which may cause participants to ignore specific intervention elements assigned to them. To avoid this issue, researchers in the VR group assist subjects in noticing the assigned elements through verbal guidance and cues. Third, the Mini-Mental State Examination (MMSE) (Tsoi et al., 2015) and MoCA (Nasreddine et al., 2005) are widely used scales that comprehensively assess cognitive function (Islam et al., 2023; Miura et al., 2024), however, they lack sensitivity to detect immediate cognitive changes. Therefore, we use the DSST and BDS scales to evaluate cognitive scores in the short-term VR intervention trial. The DSST primarily assesses working memory and processing speed, while the BDS focuses on memory capacity and attention. Although they are not as comprehensive as MMSE and MOCA, they are more sensitive to short-term cognitive changes and are widely used in cognitive assessments (Cukierman-Yaffe et al., 2020; Greendale et al., 2021; Tian et al., 2015).
